# Future direction of digital textbooks in undergraduate nursing education: A scoping review

**DOI:** 10.1371/journal.pone.0326109

**Published:** 2025-06-24

**Authors:** Aeri Jang, Hyunyoung Park, Jeong Eun Moon

**Affiliations:** 1 Department of Nursing, Mokpo National University, Muan-gun, Republic of Korea; 2 College of Nursing, Chonnam National University, Gwangju, Republic of Korea; 3 Department of Nursing, Honam University, Gwangju, Republic of Korea; Ege University, Faculty of Medicine, TÜRKIYE

## Abstract

Digital natives have emerged, and nursing educator must develop digital textbook(DT)s the meet their information processing methods. But literature reviews on DTs remain insufficient. This scoping review aims to guide nursing educators on the future direction of DTs in undergraduate nursing education. This study includes research on DTs published in English after 2016, targeting undergraduate nursing students. Two reviewers independently screened titles, abstracts, and full texts for inclusion and extracted data from Medline (PubMed), Embase (Elsevier), Cochrane Library, Cumulative Index to Nursing and Allied Health (CINAHL), and IEEE Xplore up to May 27, 2024. Of the 3,978 articles identified, 7 met the inclusion criteria. The most common study design was experimental (n = 5), and the most frequent intervention type was interactive (n = 5). The most common nursing field was caring for medically complex/special needs patients (n = 5). However, no DTs were developed for social determinants of health or mental health or for working in underserved communities. Information was provided via mobile devices (n = 2) and QR codes, applications, or links (n = 2). Six studies measured all three outcome variables: knowledge, skills, and attitudes. The technology aspects included feedback, display functions, and other technical and educational features. The findings highlight the importance of usability, integration with the nursing education curriculum, and the development of clinically based DTs for future undergraduate nursing education. Thus, DTs in undergraduate nursing education, designed for the digital native generation, should be developed based on interactive intervention-type content that facilitates easy access to personalized information and timely learning. In addition, to comprehensively cover the educational domains essential for future nurses, DTs should not only address caring for medically complex/special needs patients but also include content on social determinants of health, mental health, and nursing in underserved communities.

## Introduction

With the advancement of information and communication technology (ICT), various industries have integrated technology, bringing significant changes to education. The emergence of digital natives, also referred to as “screen people,” who grew up surrounded by mobile phones, computers, and other digital devices, has led to the adoption of visualization and gamification in information processing [1,2].

Furthermore, in response to the limitations of traditional education methods during the COVID-19 pandemic, developing innovative teaching approaches and establishing smart learning environments utilizing advanced technology has become essential in medical and nursing education [3,4]. In line with these changes, the demand and preference for digital textbooks (DTs), a type of educational technology for teaching and learning, have increased among instructors and students [5]. In addition, nursing educators are being called upon to develop DTs that align with digital natives’ information-processing methods.

A DT is a learning resource that enhances educational effectiveness by digitizing traditional printed textbooks and incorporating multimedia learning features while providing convenient functions such as search and navigation [6]. The key features of DTs include the provision of multimedia content (e.g., animations, virtual reality, videos); interactivity (e.g., discussion boards or real-time chat); and support for various learning resources (e.g., dictionaries, reference books) [7]. These features contribute to personalized learning, improved accessibility, and real-time feedback [8,9]. However, alongside physical symptoms such as eye fatigue and neck, wrist, and shoulder pain [10], limitations exist, including technostress due to information systems usage and emotional distance from learning tools [11]. Consequently, not all users prefer DTs in the classroom [12]. Therefore, for the successful development and implementation of digital textbooks, analyzing key features identified in previous studies and addressing the identified challenges are necessary.

Against this background, a scoping review was conducted to analyze existing studies on DTs in undergraduate nursing education and establish future directions for the development and implementation of DTs based on the findings.

A scoping review is a useful method for identifying the scope and trends of research on a specific topic [13] and is utilized as a research methodology for broadly mapping evidence in healthcare and education [14–16]. To date, literature reviews related to DTs include studies on the effectiveness of DT use on students’ learning outcomes in Korean primary and secondary education [7], usability elements in the DT development context [17], and health-adverse effects in using DTs [18]. However, literature reviews on DTs in undergraduate nursing education remain insufficient.

Therefore, this study aims to minimize potential trial and error developing and implementing DTs in undergraduate nursing education and provide nursing educators with effective adoption strategies. The specific research questions are as follows.

1. What are the features of DTs included in undergraduate nursing education?
2. What are the challenges of using DTs for future undergraduate nursing education?

## Materials and methods

### Protocol and registration

The study’s inclusion criteria and methods were prespecified in the Open Science Frame (https://doi.org/10.17605/OSF.IO/DHZFR). All procedures were presented according to PRISMA ScR [19], and all processes for this scoping review were prepublished in the protocol [20].

### Eligibility criteria

This study follows the Participant, Concept, Context (PCC) framework. The proposed terms selected for P are “nursing,” “nursing student,” and “undergraduate nursing education,” while the related control words are “nursing,” “students, nursing,” and “education, nursing.” The proposed term selected for Content is “digital textbook,” and the related control words are “books,” “textbook,” “monograph,” “dictionary,” “textbooks as topic,” “encyclopedia,” “reference books,” “teaching materials,” “internet,” “smartphone,” “mobile applications,” and “digital technology.” For Context, only studies targeting undergraduate nursing students were included, while data related to non-face-to-face lectures through simple video provision or examinations at nursing training institutions or other educational target, such as graduate students or nursing assistants were excluded. In addition, no geographical restrictions were applied. Specific exclusion criteria for inclusion are presented in S1 Table.

### Information sources

The study’s information sources included quantitative, qualitative, and mixed research methods; texts and opinion documents; review studies; and pilot tests. Quantitative studies, such as time series analyses, such as randomized and nonrandomized controlled trials, before-and-after studies, prospective and retrospective cohort studies, case-control studies, and longitudinal and cross-sectional technical observational studies, were excluded. Qualitative studies, including phenomenology, qualitative explanation, and behavioral research, were excluded as well. A specific table presenting the exclusion criteria will be provided. Literature published after 2016, when the Fourth Industrial Revolution was mentioned [21], was selected.

### Search

In this study, MeSH terms proposed by PCC, Emtree, and CINAHL tree, along with natural language terms, were identified and used for the final search. To prevent omitting important studies, the search scope was limited to text word searches. Based on the preliminary investigation results from prepublished Medline (PubMed) as of August 9, 2023, an additional search was conducted until May 27, 2024, after which the search strategy was established. The first search included the Embase (Elsevier), Cochrane Library, and Cumulative Index to Nursing and Allied Health (CINAHL) databases, as well as IEEE Xplore, which has numerous high-technology-related studies. In the second phase, reference lists of studies identified in the first search and citations from studies selected in systematic reviews were examined. In the third phase, grey literature was identified using ProQuest Dissertations & Theses, Google, Google Scholar, OpenGrey, and Semantic Scholar. Two reviewers independently carried out this three-step process. In cases of disagreement, issues were resolved through mutual discussion, and when consensus could not be reached, an additional reviewer participated in the revaluation to resolve discrepancies.

### Selection of source evidence

All identified studies were primarily encoded using EndNote V.20.0 (Clarivate Analytics, Pennsylvania, USA), and duplicate studies were removed. Subsequently, one reviewer (AR Jang) assessed the titles and abstracts, while two reviewers (AR Jang, JE Moon) evaluated the full text of the studies. Finally, a single reviewer (HY Park) reviewed and finalized the full text of all selected studies. Issues requiring resolution at this stage were determined through discussions among reviewers. The final list of selected references is provided in S2 Table.

### Data charting process

Two reviewers independently processed data extraction using a data extraction tool developed for this purpose. In cases of disagreement during the process, review and consultation were conducted.

### Data items

The general characteristics of the selected literature were identified based on the data extraction section of the JBI scoping review [22]. These characteristics were categorized as authors, publication year, country of origin, study aim, population and sample size, methodology, and intervention type. To address the first research question regarding the features of DTs in undergraduate nursing education, the analysis was structured into several categories: nursing field (e.g., caring for medically complex/special needs patients, population-based health, values-based care, social determinants of health, mental health, and working in underserved communities); content; information (methods of accessing DTs and their content); outcome variables; and technology/stakeholders (elements utilized by instructors and students). The study’s limitations and recommendations were analyzed to explore the future direction of DTs suitable for undergraduate nursing education.

### Synthesis of results

The study’s findings aim to propose the future direction of DTs used in undergraduate nursing education for nursing educators. The analysis results for each research question were synthesized and categorized, with the key findings summarized in the Discussion and Conclusion sections through comparative analysis with existing literature. This analysis includes identifying barriers to the advancement of DTs and proposing strategies to overcome them.

### Selection of sources of evidence

**Fig 1:**
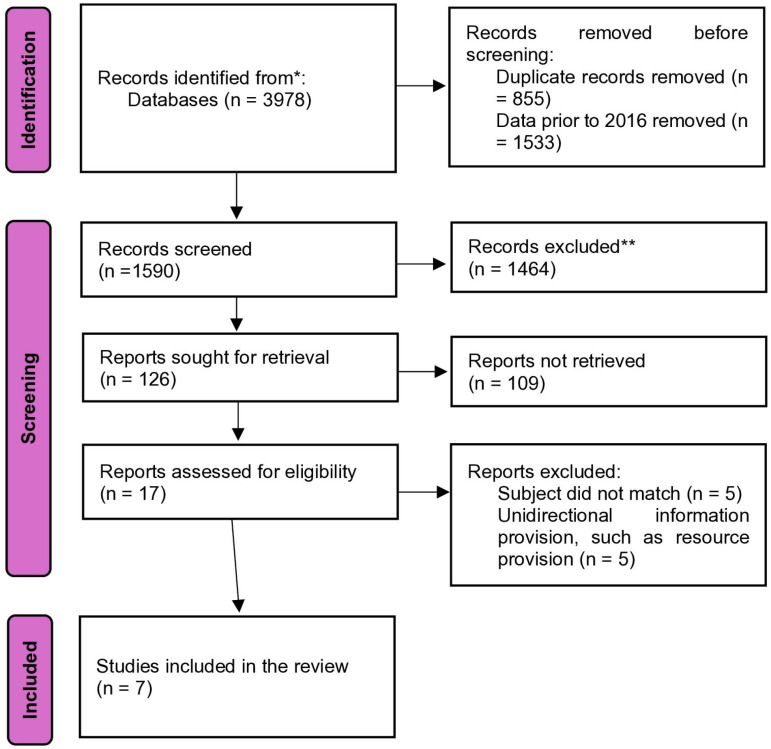
Flowchart of record retrieval and selection.

From a total of 3,978 pieces of literature, 1,590 citations were identified through searches of electronic databases. Based on the title and the abstract, 1,257 were excluded as they did not meet the eligibility criteria, leaving 30 full-text articles retrieved and assessed for inclusion. Finally, seven full-text studies were selected for inclusion in this scoping review. A PRISMA flow diagram depicting the study selection process is presented in Fig 1.

### Characteristics of sources of evidence

The year of publication showed two studies each in 2020 and 2021 and one study each in 2018, 2023, and 2024. Regarding geographical origin, Taiwan had the highest number with four studies (#1, #4, #6, #7). In terms of study types, experimental research (#1, #4–#7) was the most common with five studies, among which three (#1, #4, #7) were randomized controlled trials (RCTs). The most frequent intervention type was interactive (#1, #2, #4, #6, #7), with five studies. Detailed characteristics are presented in Table 1.

**Table 1:** General characteristics of selected studies on digital textbooks in undergraduate nursing education.

	Author (year)	Origin	Aim	Population/ Sample size (Experimental/Control)	Methodology Theoretical Framework	Intervention type (period/time)
**1**	Yu (2024)	Taiwan	To examine the effectiveness of an interactive e-book	Nursing students without prior experience in the related subject/ 62 (31/31)	Experimental Research (RCT) ARCS model	Interactive e-book (not specified)
**2**	Phillips (2023)	United States	To evaluate a new online interactive education program (OIEP)	Nursing students enrolled in Fundamental Nursing/ Instructors managing the course Student (78–81)/ Faculty (28)	Mixed methods study (Not specified)	Interactive e-book (1 semester)
**3**	Verkuyl (2021)	Canada	To develop an open educational resource e-textbook on vital sign measurement	First-year nursing students taking a health assessment course / 455	Descriptive survey, experiential teaching-learning theory	Provision of resources (2 weeks)
**4**	Chang (2021)	Taiwan	To evaluate the effects of a clinical-based sexual harassment prevention e-book	Senior nursing students / 66 (33/33)	Experimental Research (RCT) ARCS model	Provision of interactive e-book (45 min, once)
**5**	Park (2020)	South Korea	To share the process of developing digital story-based education to teach the steps of Evidence-Based Practice (EBP) and its implementation	Nurses, nursing students/ 31/91	Experimental Research (Single-group Pretest–Posttest) Evidence Based Practice learning process	Provision of an e-book for EBP-based problem-solving process (not specified)
**6**	Liu (2020)	Taiwan	To design and develop an interactive EKG-focused e-book	Nursing students enrolled in the Critical Care Nursing Course / 59 (26/33)	Experimental Research (two-group posttest design) ARCS model	Provision of interactive e-book (120 min, twice)
**7**	Sung (2018)	Taiwan	To reduce the gap between theory and practice and realize an effective learning process	Community Health Nursing Practice Student/ 87 (42/45)	Experimental Research (RCT) not mention	Provision of interactive e-book (2 weeks)

#### DT Features included in undergraduate nursing education.

In the nursing field, most studies (five studies: #1–#3, #5, #6) focused on caring for medically complex/special needs patients, while population-based health (#7) and values-based care (#4) were each covered in one study. No DTs were developed for the fields of social determinants of health, mental health, or working in underserved communities. Regarding information access methods, mobile devices (#1, #7) and QR codes/applications/links (#4, #6) were each mentioned in two studies, while email invitations (#2) and site access (#3) were each mentioned in one study. One study (#5) did not specify access methods. As for outcome variables, two studies (#5, #7) did not mention general characteristics, while others included variables such as age, gender, program participation, academic level, gender-related courses, memory ability, nursing interest, prior experience with electronic textbooks, study duration, attendance, punctuality, and overall satisfaction. In terms of effectiveness measurement, six studies (#1, #2, #4–#7) assessed participants’ knowledge, skills, and attitudes. Regarding technology/stakeholder aspects, features included feedback functions for user inquiries (#5) and content display functions on screens (#7), reflecting both technological and educational aspects. Detailed information is presented in Table 1.

#### The challenges of using DTs for future undergraduate nursing education.

**Fig 2:**
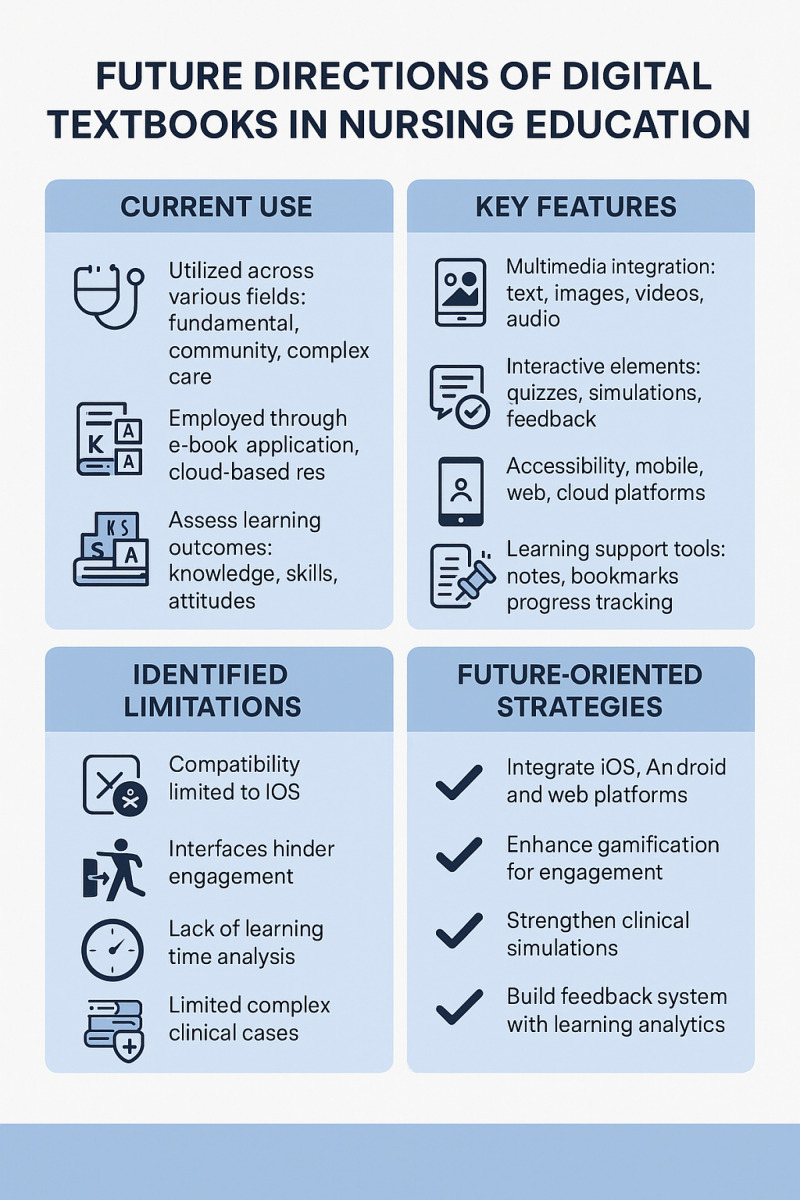
Infographic about future directions of digital textbook in nursing education.

Analysis of the studies’ limitations and recommendations highlights several key challenges. Issues related to usability, including compatibility problems (#1), the necessity of a glossary of terms (#3), and the need for technical guidance (#6) to prevent participants from feeling frustrated because of usage difficulties. In addition, recommendations emphasized the importance of integration between DTs and the nursing education curriculum, including the positive effects of integrating learning modules, such as repetitive learning and formative/summative assessments (#2), the practicality and engagement of feedback (#4), the ability to review the entire EBP process (#5), and the promotion of learning ability (#7). Moreover, the studies underscored the importance of developing clinically relevant DTs that incorporate case-based and situational problems (#3), engage users with content based on clinical situations (#4), provide experiences similar to field environments (#5), and address health-related problem resolution (#7). Specific details are presented in Table 2. All these directions are resumed on Fig 2.

**Table 2:** DT features, outcomes, and limitations in undergraduate nursing education.

	Nursing Field Contents	Outcome variable: K (Knowledge), S (Skill), A (Attitude)	Information (How to access DTs and Contents)	Technology/Stakeholders (Elements used by instructors and students)	Limitation and Recommendation
**1**	Caring for medically complex/special needs patients: Nasogastric tube feeding skill	K: Nasogastric Tube Feeding Quiz (NGFQ) S: Learning Self-Efficacy Scale (L-SES) A: Confidence Scale (C-Scale), Satisfaction Questionnaire Others: NA	Read using mobile devices (smartphone, tablet, e-book reader, etc.), which can be accessed online or downloaded for offline reading.	Integrate text, pictures, images, digital glossaries, prompts, collaborative learning methods, highlights, bookmarking, pop-up items, hyperlinks, video clips test, questions with instant feedback	The e-book developed in this study is only compatible with iOS devices, which is inconvenient. Therefore, future development should focus on creating an e-book compatible with both iOS and Android devices.
**2**	Caring for medically complex/special needs patients: Fundamental nursing	K: Cognitive Engagement Scale S: Behavioral Engagement Scale A: Affective Engagement Scale Others: Interview (Reasons for recommendation, preference, strengths, weaknesses, aspects needing change)	Email invitations were sent to students and faculty from schools using products of the creators of the OIEP.	Readings, text-to-speech listening, podcasts, simulations, animations, annotation highlighting tools, note-taking, quizzing, and test banks, practice in an online electronic health record	It was found that repetitive learning experiences and improved access to information had a positive impact on increased preference. Scaffolding and learning reinforcement activities, such as feedback on formative and summative assessments, were integrated into the module, yielding positive results. If this is widely integrated into the nursing curriculum, significant benefits will be gained.
**3**	Caring for medically complex/special needs patients: Vital sign measurement	K, S: N/A A: User-Engagement Scale (UES) Other: Practicality 12-item short form, interview (what makes an e-textbook engaging, recommendations for increasing engagement)	Can be accessed free of charge by visiting the Ryerson University Pressbooks site at pressbooks.library.ryerson.ca/vital sign/	Combines textual information, visual images, and video clips that facilitate visual, auditory, and kinesthetic learning interactive Quizzes including multiple-choice and select-all-that-apply questions, as well as documentation practice based on case studies, are offered through the open e-textbook.	A “glossary of terms” or ”key term definitions” is required to enhance engagement. Easy navigation allows for better immersion in the content. The button for moving to the next page should be more noticeable. A screen displaying progress speed is required. Various colors, text reading, and more images and diagrams are required. Rewards are needed. Quizzes and case/scenario-based questions are desired. More complex exam questions are required. Rationale for exam answers is required.
**4**	Values-based care: Sexual harassment prevention	K: Sexual Harassment Prevention Concept Scale S: Coping behavior subscale of the Sexual Harassment Scale A: Interest of participants by IMMS (Instructional Materials Motivation Survey) Others: NA	Scan the QR code to download the SimMAGIC e-book via the cloud library app on a mobile phone or tablet.	Editing and reading, importing PDF and PPT materials into editing software, integrating various multimedia components including images, photos, and text, and reading books using interactive programs. Creating one’s own e-book with interactive quizzes.	The feedback was practical and interesting. Content is engaging as it is based on clinical situations. To enhance confidence, quizzes, corrections, explanations, and repeated practice are necessary. Rewards are given for correct answers, leading to deeper learning.
**5**	Caring for medically complex/special needs patients: Patients with dementia who are complaining of pain	K, S, A: EBP questionnaire (EBPQ): cognitive load Others: Open-ended satisfaction question (no specific mention)	Not specified	Explanation of questions and consult provision function	The developed e-book allows users to review the entire process from question generation for EBP. The story character set in the background promotes empathy and immersion for learners. The developed textbook is effective in internalizing knowledge with a low cognitive load. A digital textbook based on a story similar to the clinical field allows learners to have experiences similar to those in the field environment.
**6**	Caring for medically complex/special needs patients: EKG	K: Academic achievement assessment through written exams S: N/A A: Satisfaction Other: Experiences (describe the advantages and disadvantages)	Accessing digital textbooks by reading content uploaded to the university’s e-learning system or downloading an e-book app on a computer, tablet, or smartphone.	Previewing pre-content, taking notes, providing audio explanations, and offering supplementary materials. Test: Basic and advanced challenge tests including self-assessment. Various interactive challenge tests. Feedback: Pop-up messages providing immediate feedback when students input correct content. Repetitive learning: Users can repeatedly practice cover-up and challenge tests as needed.	As learning is done through downloads, it is difficult to verify the actual online learning time. To prevent early participants from feeling frustrated, the instructor should pay attention to technical issues.
**7**	Population-based health community nursing	K: Learning effectiveness (final examination to evaluate learning achievements) S: Case presentation (intra-team, inter-team, and expert evaluation methods) A: Cognitive process: (Bloom’s taxonomy-based analysis through video recording) Other: N/A	Students used only their mobile devices with the e-book learning system.	Display course content on a screen, page flipping, bookmarking, return, and other basic functions, as well as tools to take notes that are recorded electronically. The pens can be adjusted for color, thickness, and effects. The note function includes recording sound and taking pictures, enabling students to record instructional content and capture essential points or images for later review.	E-books can enhance nursing students’ comprehension and learning abilities. They encourage self-expression, foster self-directed learning, and create an active learning environment. They facilitate interaction between teachers and students. In the future, they should be integrated not only into community practice but also into addressing and improving various health-related issues.

### Ethical considerations

The Institutional Review Board (IRB) of Nambu University, Republic of Korea, approved the exemption of this study for IRB review (1041475-2022-HR-009). Study results will be disseminated through peer-reviewed journals.

## Discussion

This scoping review aimed to address questions regarding the characteristics of DTs, the barriers encountered in their development and utilization, and the strategies needed to overcome these challenges in future undergraduate nursing education. Based on the protocol by Jang and Park [20], literature on DTs in nursing published since 2016 was searched and analyzed. Considering key components of DTs, such as learning support using animation and 3D technology, the ability to search and provide new learning materials through updates, and the interaction between synchronization and desynchronization [8], findings revealed that literature on DT development in undergraduate nursing programs has not been actively produced over the past decade. These findings align with observations by Jang and Park [20], who noted a lack of systematic reviews on DTs used in nursing education. The effectiveness of DTs in qualitative education [23] and the growth of e-textbooks in the education sector [24] have already been established, and the demand for DT development and dissemination has accelerated because of the COVID-19 pandemic. However, the limited development of DTs in undergraduate nursing programs may be attributed to the nature of nursing education, which is not a theoretical discipline where learning can be induced solely through web-based content consumption by observers or readers. Instead, nursing is a practice-oriented field where students learn by performing procedures, generating questions, reviewing, and verifying their understanding. Rather than focusing on the development of DTs, there has likely been a greater emphasis on developing and applying simulation-based learning content, such as virtual simulation, mixed reality (MR), and extended reality (XR).

Upon reviewing the final selection of seven studies, the most common intervention type was interactive. This focus reflects the rapid expansion and transformation of Internet-based industries across society, which has also driven educational changes. As a result, “learner-centered education,” one of the core areas of teaching and learning, has also been incorporated into DTs [25]. Learners can develop active, self-directed learning competencies and create value independently. To facilitate the provision of personalized learner information and just-in-time learning, it is deemed essential for the development of future nursing DTs to advance in the direction of intervention-type approaches.

Regarding the nursing fields covered in DTs, most studies focused on caring for medically complex/special needs patients. In contrast, population-based health and values-based care were each represented by only one study. This analysis is based on the key competencies that future nurses should develop, as outlined by the National Academy of Sciences [26], and aligns with findings from previous literature. However, no development of DTs has been addressing social determinants of health, mental health, or working in underserved communities. This gap likely exists because hospital-based patient care occupies a larger portion of the nursing education curriculum than social factors, underserved populations, or mental health topics. Future research should focus on addressing these underdeveloped areas.

This study compares and analyzes general characteristic variables and effect evaluation variables. General characteristics, memory ability, nursing interest, prior experience with electronic textbooks, subject-related study duration, attendance, punctuality, overall satisfaction, and aspects presented in the technology/stakeholder category were referenced to guide researchers attempting to develop and study DTs in the future. Shorey et al. [27] stated that for current and future generations of learners, referred to as digital natives, DT can be an alternative for reading numerous paragraphs and structuring their knowledge through new questions. Therefore, for DTs to be well-established and actively developed as the next-generation learning content in nursing education, continuous development and research should consider the characteristics of learners using DT and the variables that can assess its effectiveness.

Through the selected literature, this review aimed to identify the future direction of DTs. The selected literature discusses usability (#1, #3, #6), the integration of DTs with the nursing education curriculum (#2), and the importance of developing clinical-based DTs (#3–#5, #7). Reid et al. [2] emphasized that digital literacy, which includes efficiently searching for and critically evaluating information while recognizing the risks of biased sources, should be incorporated into undergraduate nursing curricula. In addition, Ye [5] identified interactivity, reading convenience, ease of use, and searchability as factors influencing the perceived suitability of digital textbooks, which serve as the basis for students evaluating DTs as interactive online learning tools. This finding aligns with the characteristics of DTs identified in the studies analyzed in this research. These findings provide a foundation for determining the future direction of DTs. Given that young digital natives [2], who have been surrounded by mobile phones, computers, and other digital devices throughout their lives, are the primary audience of undergraduate nursing education and the future nursing workforce, nursing education must consider their characteristics and the resources they primarily use for learning. Nursing services must also adapt to societal changes, necessitating the development of on-demand learning content that meets learners’ diverse levels and needs and fostering global community collaboration and cooperation [4]. However, due to the increasing emphasis on patient safety in healthcare settings, undergraduate nursing students face restrictions on direct patient care during clinical training, leading to an observation-based approach. Therefore, nursing education must reflect the characteristics and needs of learners while incorporating experiential learning, problem-solving education, and empathy-based education. In addition, curricula should be developed and actively utilized based on DTs that enable real-time access to, critical evaluation of, and integration of live information reflecting the rapidly evolving clinical environment. Finally, the direction that digital textbooks should take in future nursing education, as suggested in the infographic in Fig 2, is to standardize the platform and move toward a form that allows for fun and feedback based on clinical situations. This implies that it should be developed into a standardized platform that allows for practice-based interaction beyond the existing theory and practice stages.

This study excluded qualitative research and literature published before 2016 when the Fourth Industrial Revolution was officially mentioned. In addition, this study excluded literature related to DTs for graduate students or nurses and focused solely on digital textbooks used as part of undergraduate nursing curricula. Therefore, as this study does not provide a comprehensive analysis of all digital textbooks developed and utilized in undergraduate nursing programs, there may be limitations in applying the findings.

## Conclusion

Given that nursing is a practice-oriented discipline, the rapid pace of change in high-tech-based clinical practice and learners from the generation referred to as digital natives suggest that nursing education can no longer emphasize text-based learning alone. Therefore, DTs for future nursing education should be designed to facilitate easy retrieval of learner-customized information and enable timely learning through interactive features. They should be developed based on intervention-type content. In addition, to comprehensively cover the educational domains that future nurses must master, these DTs should include nursing care for medically complex or special needs patients and topics such as social determinants of health, mental health, and nursing in underserved areas. This suggests the need to reconsider the traditional theory-practice separation paradigm in nursing education and highlights the importance of systematically developing and researching DTs as a key strategy to enhance the quality of future nursing education and support learner-centered instruction.

## Supporting information

S1 Table Inclusion and exclusion criteria. (DOCX)

S2 Table List of selected studies. (DOCX)

## References

[1] Prensky M. Digital natives, digital immigrants part 1. Horizon. 2001;9(5):1–6. doi: 10.1108/10748120110424816

[2] Reid L, Button D, Brommeyer M. Challenging the myth of the digital native: a narrative review. Nurs Rep. 2023;13(2):573–600. doi: 10.3390/nursrep13020052 37092480 PMC10123718

[3] Buckley H. Faculty development in the COVID-19 pandemic: So close - yet so far. Med Educ. 2020;54(12):1189–90. doi: 10.1111/medu.14250 32436272 PMC7280722

[4] Oh EG. Perspectives on nursing profession for a post-COVID-19 new normal. Korean J Adult Nurs. 2020;32(3):221–2. doi: 10.7475/kjan.2020.32.3.221

[5] Ye C. Antecedents and consequences of perceived fit of an interactive digital textbook. JISE. 2021;2:27–39.

[6] Kim JH-Y, Jung H-Y. South Korean digital textbook project. Comput Sch. 2010;27(3–4):247–65. doi: 10.1080/07380569.2010.523887

[7] Jang D-H, Yi P, Shin I-S. Examining the effectiveness of digital textbook use on students’ learning outcomes in South Korea: A meta-analysis. Asia Pacific Edu Res. 2015;25(1):57–68. doi: 10.1007/s40299-015-0232-7

[8] Joo YJ, Park S, Shin EK. Students’ expectation, satisfaction, and continuance intention to use digital textbooks. Comput Hum Behav. 2017;69:83–90. doi: 10.1016/j.chb.2016.12.025

[9] Roberts K, Benson A, Mills J. E-textbook technology: Are instructors using it and what is the impact on student learning? JRIT. 2021;14(3):329–44. doi: 10.1108/jrit-04-2021-0028

[10] Seomun G, Lee J-A, Kim E-Y, Im M, Kim M, Park S-A, et al. Health effects of digital textbooks on school-age children: a grounded theory approach. West J Nurs Res. 2013;35(9):1184–204. doi: 10.1177/0193945913491838 23780942

[11] Tarafdar M, Cooper CL, Stich J. The technostress trifecta ‐ techno eustress, techno distress and design: Theoretical directions and an agenda for research. Inf Syst J. 2017;29(1):6–42. doi: 10.1111/isj.12169

[12] Verkijika SF. Digital textbooks are useful but not everyone wants them: The role of technostress. Comput Educ. 2019;140:103591. doi: 10.1016/j.compedu.2019.05.017

[13] Arksey H, O’Malley L. Scoping studies: towards a methodological framework. Int J Soc Res Methodol. 2005;8(1):19–32. doi: 10.1080/1364557032000119616

[14] Khalil H, Peters M, Godfrey CM, McInerney P, Soares CB, Parker D. An evidence-based approach to scoping reviews. Worldviews Evid Based Nurs. 2016;13(2):118–23. doi: 10.1111/wvn.12144 26821833

[15] Maggio LA, Larsen K, Thomas A, Costello JA, Artino AR Jr. Scoping reviews in medical education: A scoping review. Med Educ. 2021;55(6):689–700. doi: 10.1111/medu.14431 33300124 PMC8247025

[16] Munn Z, Peters MDJ, Stern C, Tufanaru C, McArthur A, Aromataris E. Systematic review or scoping review? Guidance for authors when choosing between a systematic or scoping review approach. BMC Med Res Methodol. 2018;18(1):143. doi: 10.1186/s12874-018-0611-x 30453902 PMC6245623

[17] Wan Sulaiman WNA, Mustafa SE. Usability elements in digital textbook development: a systematic review. Pub Res Q. 2019;36(1):74–101. doi: 10.1007/s12109-019-09675-3

[18] Seomun GA, Kim EY, Noh WJ. A review of studies on the health-adverse effects in using digital textbooks. J Digit Converg. 2012;10(1):165–75. doi: 10.14400/JDPM.2012.10.1.165

[19] Tricco AC, Lillie E, Zarin W, O’Brien KK, Colquhoun H, Levac D, et al. PRISMA extension for scoping reviews (PRISMA-ScR): checklist and explanation. Ann Intern Med. 2018;169(7):467–73. doi: 10.7326/M18-0850 30178033

[20] Jang A, Park H. Digital textbooks for undergraduate nursing education: a scoping review protocol. BMJ Open. 2024;14(7):e071147. doi: 10.1136/bmjopen-2022-071147 39013648 PMC11331992

[21] World Economic Forum. 2016 World Economic Forum Annual Meeting. 2016 Jan 20–23 [cited 3 Sep 2021]. Available from: https://www.weforum.org/events/world-economic-forum-annual-meeting-2016/programme

[22] Aromataris E, Lockwood C, Porritt K, Pilla B, Jordan Z, editors. JBI Manual for Evidence Synthesis. JBI; 2024. Available from: https://synthesismanual.jbi.global/

[23] Gaberson K, Oermann M, Schellenbarger T. Clinical teaching strategies in nursing. 4th ed. New York: Springer; 2015.

[24] Dobler E. E-textbooks: a personalized learning experience or a digital distraction? J Adolesc Adult Lit. 2015;58(6):482–91.

[25] Seo Y-S, Choi S-S, Shin S-W, Kwon Y-J, Kim K-Y, Choi M-A. Research to develop a road map for e-learning standardization. Korea Agency for Technology and Standards; 2009 [cited 16 Aug 2024]. Available from: https://www.keris.or.kr/common/fileDownload.do?fileKey=0c94afc93701ec25da6512ebcb1e2810&dwlTy=pblcte

[26] National Academies of Sciences, Engineering, and Medicine; National Academy of Medicine; Committee on the Future of Nursing 2020–2030. Educating nurses for the future. In: Flaubert JL, Le Menestrel S, Williams DR, et al., editors. The future of nursing 2020-2030: Charting a path to achieve health equity. Washington (DC): National Academies Press (US); 2021. p. 189–246. 34524769

[27] Shorey S, Chan V, Rajendran P, Ang E. Learning styles, preferences and needs of generation Z healthcare students: Scoping review. Nurse Educ Pract. 2021;57:103247. doi: 10.1016/j.nepr.2021.103247 34768214

