# Supplementary material for: Future direction of digital textbooks in undergraduate nursing education: A scoping review
Source: PLoS One. 2025 Jun 24;20(6):e0326109. doi: 10.1371/journal.pone.0326109 (PMC12186970; doi:10.1371/journal.pone.0326109)
Supplement: S1 Table — (DOCX) [file pone.0326109.s001.docx]

| **Inclusion Criteria** | **Exclusion Criteria** |
| --- | --- |
| - Studies published from 2016 onward - English-language publications only - Involving nursing students - Digital textbooks that integrate both text and interactive modules - Programs aimed at enhancing clinical skills as alternatives to practice | - Studies that simply provide resources without instructional structure - Surveys, toolkits, or protocol development studies - Studies using only pre-learning videos - Review articles or concept analysis studies - Bibliometric analyses - Educational model development studies - Video lectures without interactive content - Opinion papers without empirical support on future directions - Conference abstracts without full-text availability - Studies where the full text is not retrievable - Printed textbooks converted to online formats without digital enhancement |
