# Supplementary material for: Future direction of digital textbooks in undergraduate nursing education: A scoping review
Source: PLoS One. 2025 Jun 24;20(6):e0326109. doi: 10.1371/journal.pone.0326109 (PMC12186970; doi:10.1371/journal.pone.0326109)
Supplement: S2 Table — (DOCX) [file pone.0326109.s002.docx]

| 1. Yu T-Y, Huang T-W, Huang H-C, Li S-Y, Chuang Y-H. Effects of an interactive e-book on enhancing nursing students’ knowledge, confidence, and learning self-efficacy of nursing skills: a randomized controlled trial. Nurse Educ. 2024;49(1): E20–E25. doi:10.1097/NNE.0000000000001490 |
| --- |
| 1. Phillips BC, Johnson J, Khalid N, Zapparrata N, Albright G. Benefits of an online interactive educational program over traditional textbooks. Nurse Educ. 2023;48(5): 270–275. doi:10.1097/nne.0000000000001398 |
| 1. Verkuyl M, Atack L, Lapum JL, Hughes M, St-Amant O, Petrie P. User engagement using an etextbook: a descriptive study. Comput Inform Nurs. 2021;39(11): 668–674. doi:10.1097/CIN.0000000000000720 |
| 1. Chang T-S, Teng Y-K, Chien S-Y, Tzeng Y-L. Use of an interactive multimedia e-book to improve nursing students’ sexual harassment prevention knowledge, prevention strategies, coping behavior, and learning motivation: a randomized controlled study. Nurse Educ Today. 2021;105: 104883. doi:10.1016/j.nedt.2021.104883 |
| 1. Park M, Jeong M. Digital story-based education: an innovative way to learn evidence-based practice. J Contin Educ Nurs. 2020;51(11): 501–503. doi:10.3928/00220124-20201014-05 |
| 1. Liu Y, Chou P-L, Lee B-O. Effect of an interactive e-book on nursing students’ electrocardiogram-related learning achievement: a quasi-experimental design. Nurse Educ Today. 2020;90: 104427. doi:10.1016/j.nedt.2020.104427 |
| 1. Sung T-W, Wu T-T. Learning with e-books and project-based strategy in a community health nursing course. Comput Inform Nurs. 2018;36(3): 140–146. doi:10.1097/cin.0000000000000398 |
